# Transition and Damping of Collective Modes in a Trapped Fermi Gas between BCS and Unitary Limits near the Phase Transition

**DOI:** 10.1038/srep15848

**Published:** 2015-11-02

**Authors:** Hang Dong, Wenyuan Zhang, Li Zhou, Yongli Ma

**Affiliations:** 1 Surface Physics Laboratory and Department of Physics, Furan University, Shanghai 200433, China

## Abstract

We investigate the transition and damping of low-energy collective modes in a trapped unitary Fermi gas by solving the Boltzmann-Vlasov kinetic equation in a scaled form, which is combined with both the *T*-matrix fluctuation theory in normal phase and the mean-field theory in order phase. In order to connect the microscopic and kinetic descriptions of many-body Feshbach scattering, we adopt a phenomenological two-fluid physical approach, and derive the coupling constants in the order phase. By solving the Boltzmann-Vlasov steady-state equation in a variational form, we calculate two viscous relaxation rates with the collision probabilities of fermion’s scattering including fermions in the normal fluid and fermion pairs in the superfluid. Additionally, by considering the pairing and depairing of fermions, we get results of the frequency and damping of collective modes versus temperature and *s*-wave scattering length. Our theoretical results are in a remarkable agreement with the experimental data, particularly for the sharp transition between collisionless and hydrodynamic behaviour and strong damping between BCS and unitary limits near the phase transition. The sharp transition originates from the maximum of viscous relaxation rate caused by fermion-fermion pair collision at the phase transition point when the fermion depair, while the strong damping due to the fast varying of the frequency of collective modes from BCS limit to unitary limit.

**S**trongly-interacting two-component Fermi gases provide a unique testing ground for the many-body theories of exotic systems, such as unconventional superconductors, nuclear matter, neutron stars and dilute atomic Fermi gases, which at first sight have tunable and strong interactions by using a Feshbach resonance (FR)^1^,^2^. Near the resonance [*η* ~ 0, as usual, we conveniently measure the interaction strength in terms of the inverse scattering length *η* = (*k*_*F*_*a*_*sc*_)^−1^, where *k*_*F*_ is the Fermi momentum and *a*_*sc*_ the *s*-wave scatting length], the interparticle interactions are unitary limited and universal^1^,^2^,^3^,^4^.

The study of collective excitations in these systems has attracted much attention in the past decades. The collective excitations are one of the main sources to prob the dynamics of the many-body systems. The high accuracy of frequency measurements and the sensitivity of collective phenomena to interaction effects make them good candidates to unravel the dynamical correlations. Experimental results have been obtained for low-lying collective modes of a two-component Fermi gas ^6^Li in wide temperature and interaction regimes, including the radial compression modes^5^,^6^,^7^,^8^,^9^,^10^, the axial compressions modes^8^,^11^,^12^, the radial quadrupole modes^10^,^13^,^14^, and the scissors mode^10^,^13^. The thermodynamic quantities like energy and entropy in trapped Fermi gases at unitarity are measured without invoking any specific theoretical model^15^,^16^. These experiments have in turn stimulated a considerable amount of theoretical works^11^,^12^,^17^,^18^,^19^,^20^,^21^,^22^. However, a theoretical description of this unitary regime is still challenging, particularly at nonzero temperature for sharp transition and strong damping of the collective modes.

There are different strong-coupling theories to study the collective excitations of superfluid Fermi gases in the BCS-BEC crossover. One of them is microscopic theory based on a model Hamiltonian either with a one-channel model for a broad (or weak) FR or with a two-channel model for a narrow FR. The link of these two models is well described in^23^. There are numerous efforts to develop the strong-coupling perturbation theories of interacting fermions. For example, the thermodynamic potential (or action) approach^24^,^25^,^26^,^27^,^28^, the diagrammatic method^29^,^30^,^31^,^32^,^33^,^34^,^35^,^36^,^37^,^38^,^39^, and the many-body *T*-matrix fluctuation theories^29^,^30^,^31^,^32^,^33^,^34^,^35^,^36^,^37^,^38^,^39^,^40^,^41^,^42^,^43^,^44^. Leggett’s^45^ mean-field theory and then Randeria *et al.*^46^ by adding fluctuations get some qualitative correct results at zero temperature. The Quantum Monte Carlo (QMC) simulations^47^,^48^ and the pseudogap approach^43^,^49^,^50^,^51^ have better results in the BCS-BEC crossover. Because the strong coupling atomic Fermi gases are trapped in a finite space at finite temperatures, the inhomogeneous feature of the system, strong pairing fluctuations, and finite temperatures are important keys in considering real cold Fermi gases, which makes the pure microscopic approach difficult to deal with, especially in studying the collective excitations.

Another viewpoint is the quantum hydrodynamical theory based both on the Boltzmann-Vlasov kinetic equation in the normal state^17^,^18^,^19^,^20^,^21^,^22^ and on the generalized Gross-Pitaevskii(GP) equation in the superfluid state^52^,^53^,^54^,^55^,^56^,^57^,^58^,^59^,^60^,^61^,^62^ with a phenomenological equation of state. The study of the viscosity of strongly interacting systems is also a topic of great interest both in experimental works^5^,^6^,^7^,^8^,^9^,^10^,^11^,^12^,^13^,^14^,^63^,^64^ and in theoretical works based on the Boltzmann-Vlasov kinetic equation^17^,^18^,^19^,^20^,^21^,^22^,^65^,^66^. The radial compression mode reveals a surprising behavior^8^: An abrupt change of the radial collective frequency in a strongly attractive Fermi gas. The radial quadrupole mode has confirmed the transition from collisionless to hydrodynamic behavior at 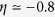
14. The transition is accompanied by very strong damping. The corresponding features cannot be explained on the basis of available theoretical models and new physics is in great need in this regime. How to explain this feature is an open question by now. We still lack a full discussion on the transition and damping of collective modes, especially compared with the experimental results^5^,^6^,^7^,^8^,^9^,^10^,^11^,^12^,^13^,^14^,^63^,^64^. This is the major motivation for our present study of different collective modes under similar experimental conditions where the system is trapped around the critical temperature *T*_*c*_.

In this paper, we determine the sharp transition and strong damping of the collective modes at −2 < *η* < 0 around *T*_*c*_ after solving the Boltzmann-Vlasov kinetic equation in a scaled form, in which we have combined both the many-body *T*-matrix fluctuation theory in the normal phase at *T* ≥ *T*_*c*_ and the mean-field theory in the order phase at *T*, *T*_*c*_. We first need to get the expressions of the viscous relaxation time, *τ*_*nn*_ and *τ*_*ns*_. Here *τ*_*nn*_ is related to the scattering between fermions in the normal states, and *τ*_*ns*_ is related to the scattering between normal fermions and superfluid fermions. In order to do so, the two-fluid approach is a link to the microscopic and kinetic descriptions of many-body Feshbach scattering. We then calculate two viscous relaxation rates by solving the Boltzmann-Vlasov steady state equation in a variational form. We next calculate the collective-mode frequencies and their damping as a function of the temperature and interaction strength for a trapped gas. We finally compare our results to experiments.

## Results

### Model and ingredients

For the broad resonance regime of the interacting fermions, a one-channel microscopic model of the strong Feshbach resonance is the most appropriate. For *N* fermions of two species *σ* = ↑ and *σ* = ↓ with fermionic field *ψ*_*σ*_(**r**), its Hamilton density is


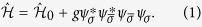


Here 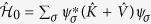 is the single-particle Hamilton density, 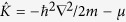, 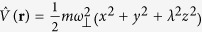 is the external potential with *λ* being the anisotropic parameter, and *g* = 4*πħa*_*sc*_/*m* is the interaction coupling constant. In the normal phase at *T*  ≫ *T*_*c*_, the system can be treat as a normal fluid. But near above *T*_*c*_, fermions in the system may exist pre-pairing. While in the order phase at *T* < *T*_*c*_, parts of fermions become either the Cooper pairs due to the many-body effect at the BCS side or the bosonic molecule due to two-body bound state at the BEC side, and the pair fluctuations become important to the system near the unitary. However, it is hard to find anact solution of the model to describe this picture, also hard to find the expressions for the viscous relaxation rates of the all scattering processes.

Firstly, we start from the Boltzmann-Vlasov kinetic equation and find its scaling solutions. The phenomenological two-fluid physical picture is about *N*_*n*_ normal fermions and *N*_*s*_ = (*N* − *N*_*n*_)/2 superfluid fermion pairs (Cooper pairs or molecules). Generally, we should write GP equation to describe the dynamic of the superfluid parts. However, our interest focus on the system’s behavior and novel phenomenon near *T*_*c*_, where the effects that form and break pairs play a more important role. So we freeze all superfluid fermion pairs at zero center-mass momentum and omit the dynamics of the superfluid fermions. This approximation is only satisfied at the BCS side. Therefore we focus on two kinds of scattering processes of fermions from BCS limit to unitary limit. One is scattering with the normal fermions in the normal phase by joining the *T*-matrix approximation at *T* ≥ *T*_*c*_, the other is with the fermion pairs in the superfluid phase by adopting the mean-field approximation at *T* < *T*_*c*_. Secondly, in order to consider the pairing and depairing effects of fermions, and to get the pair-pair and fermion-pair coupling constants in the order phase, we need to connect the microscopic and kinetic descriptions of many-body Feshbach scattering. So we solve the one-channel microscopic model in the mean-field approximation. We write the two-liquid phenomenological model and determine the coupling constants by equalizing all the physical quantities obtained from the phenomenological model with those obtained from the microscopic model. Thirdly, we derive the expression of the two viscous relaxation time of fermions, and calculate their relaxation time, *τ*_*nn*_ and *τ*_*ns*_, respectively. Finally, we calculate the collective mode frequencies and their damping as a function of the temperature and interaction strength for a trapped gas. We compare our theoretical results to experiments, and conclude that the transition from collisionless to hydrodynamic behavior occurs at the maximum of viscous relaxation rate of normal fermion-superfluid pair collision at phase transition point.

### Boltzmann-Vlasov kinetic equation

We first briefly review the Boltzmann-Vlasov kinetic equation. We consider a two-component gas of Fermi atoms with mass *m* and different spin *σ* near its normal phase. We assume that the dynamics is described by a semiclassical distribution function *f*(**r**, **p**, *t*) for each component. We omit the index of spin because of the system symmetry. *f*(**r**, **p**, *t*) satisfies the Boltzmann-Vlasov kinetic equation^18^,^67^,^68^


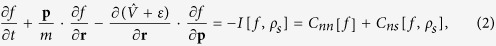


where *C*_*nn*_[ *f*] and *C*_*ns*_[ *f*, *ρ*_*s*_] are the collision integrals of normal fermion-normal fermion and normal fermion-superfluid pair, respectively. They are a functional of *f*(**r**, **k**, *t*) and density of pairs *ρ*_*s*_(**r**) near below *T*_*c*_. **k** = **p**/*ħ*, and *ħ* = *h*/2*π* with *h* being the Plank constant. *ε* is the mean-field interaction energy. Many physical quantities are very sensitive to the equation of state which is given by 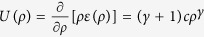 in a polytropic approximation *ε*(*ρ*) = *cρ*^*γ*^ with *c* being the constant with given *η*. Here *γ* is an index defined as the logarithmic derivative *γ* = ∂ *ln ε*/∂ *ln ρ*^55^, and *ρ* is the particle density for each component of Fermi gas. Interparticle interactions enter Eq. (2) in two different ways. One way is to modify the effective potential through the mean-field term *ε* which affects the streaming part of the Boltzmann kinetic equation. The mean-field term is essential linear in *a*_*sc*_ and this theory has no dissipative term. Another way is to consider the two-body interactions in the collision integral −*I*[ *f*, *ρ*_*s*_] which is quadratic in the scattering length and describes the dissipative processes.

The two collision terms in Eq. (2) at Born approximation level are given by^67^,^68^


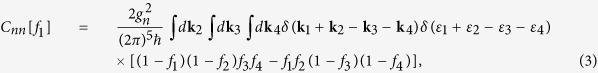



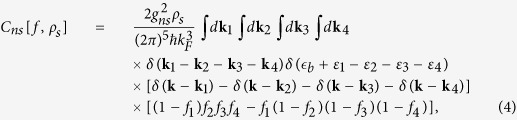


with *f*_*i*_  ≡  *f*(**r**, **k**_*i*_, *t*) for *i* = 1, 2, 3, 4. Here *g*_*n*_ = *g* = 4*πħa*_*sc*_/*m*. *g*_*ns*_ is the fermion-pair coupling constant determined below. The binding energy 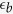 of a fermion pair is always negative below *T*_*c*_.

In order to study the damping rate, we need to calculate the viscous relaxation rate 1/*τ* = 1/*τ*_*nn*_ + 1/*τ*_*ns*_. In the normal phase at *T* ≥ *T*_*c*_, *τ* = *τ*_*nn*_ as usual, while in the superfluid phase at *T* < *T*_*c*_, we have to estimate *τ*_*ns*_. We first write *f* = *f  *^0^ + *δf* where 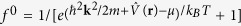 is the equilibrium distribution function without the interactions between two particles and *δf* is its deviation with the interactions. The Boltzmann-Vlasov kinetic equation (2) is a differential and integral equation, and it is hard to find its solution exactly. In the relaxation time approximation, *I*[Φ] = ( *f* − *f  *^0^)/*τ* where Φ is defined by *δf* = *f  *^0^(1 − *f  *^0^)Φ. So


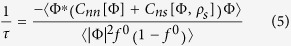


for any trial function Φ by using a variational method. Here 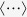 means an integral over all phase space (both coordinate and momentum space), i.e., 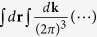. We will model the scatting process and calculate Eq. (5) below.

### The scale solutions of Boltzmann-Vlasov kinetic equation

We investigate the low-lying collective oscillations with both the mean-field and the dissipative contributions by means of the scaling factor method. We take ansatz^17^,^69^


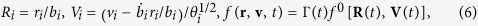


where 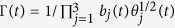. The particle density takes the form of 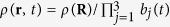. The dependence on time is contained in the parameters *b*_*i*_ and *θ*_*i*_. Following refs 17,69, we substitute this ansatz into Eq. (2), integrate in phase space, and calculate the average moment of *R*_*i*_*V*_*i*_. This leads to


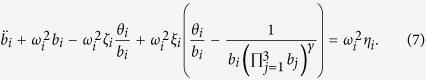


Here


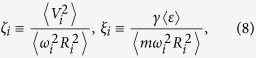


and


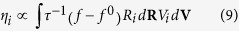


does not vanish at *T* ≤ *T*_*c*_ but is hard to calculate directly. The average means in the equilibrium density of *ρ*(**R**) within the atom cloud. The average moment of 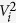 leads to


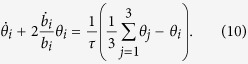


Eq. (10) depends explicitly on the viscous relaxation time 1/*τ* = 1/*τ*_*nn*_ + 1/*τ*_*ns*_.

By linearizing the Eqs. (7) and (10) around equilibrium (*b*_*i*_ = *θ*_*i*_ = 1) which gives the generalized Virial theorem of 1 − *ζ*_*i*_ − *ξ*_*i*_ = *η*_*i*_ with *i* = (⊥, *z*) in the axial symmetry external potential, we find the following dispersion law


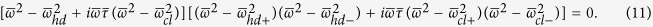


Here subscripts *hd* and *cl* denote the hydrodynamic (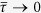) and collisionless (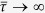) regimes, respectively. And


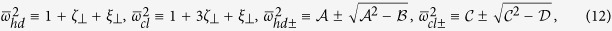



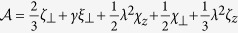
, 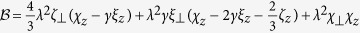 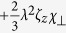, 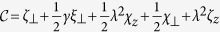, 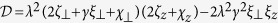, with *χ*_⊥_ = *ζ*_⊥_ + (*γ* + 1)*ξ*_⊥_ + 1 and *χ*_*z*_ = *ζ*_*z*_ + (*γ* + 1)*ξ*_*z*_ + 1. When we study the collective modes in the absence of the interacting term of *ε* but with the dissipative contribution (*ξ*_*i*_ = 0 and *ζ*_*i*_ = 1 at *T* ≥ *T*_*c*_, the collision integral represents the two-body interactions), Eq. (12) becomes 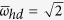, 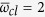, 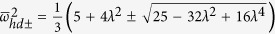, 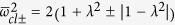. The mode frequency and damping are sensitive to the dimensionless viscous relaxation rate 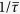 as a function of (*T*, *η*, *λ*, *N*). For example, the equation from the first factor of the left-hand side of Eq. (11) gives the quadrupole mode frequencies in the transverse directions. This equation is the same with the one obtained early in refs 69, 70, 71. It is clear that the transition between the collisionless (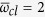) and hydrodynamic (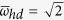) modes is determined by the behavior of the dimensionless viscous relaxation time 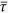. The damping exists due to the finite value of 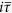 in those equations. The second factor of the left-hand side of Eq. (11) gives the frequencies of the two compression modes labeled by + and − as the radial and axial modes, respectively. Thus, our work is to determine *ξ*_*i*_ and *ζ*_*i*_ (*i* = ⊥, *z*), and 
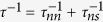
, as functions of (*T*, *η*, *λ*, *N*) near the phase transition. This extends the results in refs 69, 70, 71 and allows a direct comparison with experiments. To get these quantities, we adopt the *T*-matrix approximation at *T* ≥ *T*_*c*_ and the mean-field approximation at *T* < *T*_*c*_.

### *T*-matrix fluctuation theory above the critical temperatures *T*_*c*_

For a trapped system, we use the local density approximation (LDA) and write the chemical potential as 
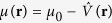
 where *μ*_0_ is chemical potential at the center of trap. *μ* is determined by *N* = ∫*d***r***ρ*(**r**). The total potential energy is 
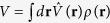
. To find a expression for *ρ*(**r**) and discuss the strong-coupling effects in the BCS-BEC crossover above *T*_*c*_, we include pairing fluctuations within the *T*-matrix approximation^29^,^30^,^31^,^32^,^33^,^34^,^35^,^37^,^38^,^39^,^40^,^41^,^42^,^43^,^44^. In ladder approximation, the single-particle thermal Green’s function is 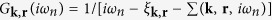, where *ω*_*n*_ = (2*n* + 1)*π*/*β* is the fermion Matsubara frequency for 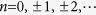, *ξ*_**k**,**r**_ = *ε*_**k**_ − *μ*(**r**), *ε*_**k**_ = *ħ*^2^**k**^2^/2*m*, and 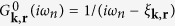 is the free fermion Green’s function. The self-energy correction 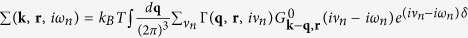 describes effects of pairing fluctuations, where *ν*_*n*_ = 2*nπ*/*β* is the boson Matsubara frequency and *δ* is the infinitesimal positive constant. The particle-particle scattering matrix is given by


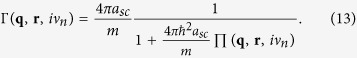


This describes the fluctuations in the Cooper channel. Here Π(**q**, **r**, *i ν*_*n*_) is the pair propagator expressed as


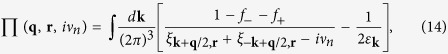


with 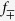 being the equilibrium Fermi distribution function *f  *^0^ for 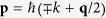 in the absence of the inter-particle interactions. The superfluid phase transition temperature *T*_*c*_ is given as the temperature at which the Thouless criterion^72^ is satisfied in the trap center (*r* = 0). The resulting *T*_*c*_ equation is given by Γ^−1^(**q** = 0, **r** = 0, *iν*_*n*_ = 0) = 0.

We now calculate *μ*, *ξ*_*i*_ and *ζ*_*i*_ for *i* = (⊥, *z*) at *T* > *T*_*c*_. For the harmonic trap within LDA, 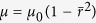 under the Thomas-Fermi approximation with the dimensionless spherical polar coordinate 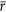 in the radial direction. At *T* > *T*_*c*_, the chemical potential *μ* is determined by the condition


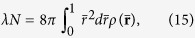


and the particle density 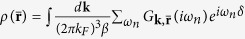. One of our calculations of *μ* is identical to the one described in refs 40,41, and another is the numerical Fourier transformation similar with refs 30,39 where more details are given, and the resulting multidimensional integrals are evaluated by using a Monte Carlo routine. The mean potential energy is always determined by


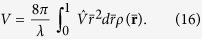


At high enough temperature *T * ≫ *T*_*c*_, the mean kinetic energy is *K* = 3*Nk*_*B*_*T*/2 and the mean interaction energy *U* is determined by the Virial theorem: *ξ*_*i*_ = 1 − *ζ*_*i*_. At *T* ≥ *T*_*c*_, *U* is determined by the definition from BCS limit to unitary limit


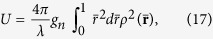


and *K* is determined by the Virial theorem: *ζ*_*i*_ = 1 − *ξ*_*i*_. Therefore, we can obtain *ξ*_*i*_ and *ζ*_*i*_ in whole BCS side of BCS-BEC crossover at *T* ≥ *T*_*c*_.

### Mean-field theory below *T*_*c*_

We choose the mean-field theory to describe the system below *T*_*c*_. In order to calculate the viscous relaxation rate 1/*τ*_*ns*_ and ratios *ξ*_*i*_ and *ζ*_*i*_ for *i* = (⊥, *z*), we express the fermion-pair and pair-pair coupling constants *g*_*ns*_ and *g*_*s*_ by combining the mean-field microscopic theory with the two-fluid approach that the system consists of *N*_*n*_ normal fluid fermions and *N*_*s*_ superfluid fermion pairs at *T* < *T*_*c*_. On the one hand, we calculate the physical quantities within the mean-field theory, such as the particle number density, chemical potentials, energy gap, excitation energies, and the total energy. On the another hand, the two-fluid approach gives the effective energy *H*_*eff*_ = *K* + *V* + *U*, where *K*, *V* and *U* are the kinetic energy, potential energy and interacting energy, respectively. We make a connection between *H*_*eff*_ and *A* in the Leggett’s mean-field theory to make sure they describe the same system at the level of *E*, *μ*, and *ρ*(**r**). Thus we can obtain the expressions for *g*_*ns*_, *g*_*s*_, and *ρ*_*s*_(**r**) in this approach. Meanwhile, we can obtain the ratios *ξ*_*i*_ and *ζ*_*i*_ for *i* = (⊥, *z*) in this way.

Firstly, the one-channel microscopic theory with mean-field approximation gives the action


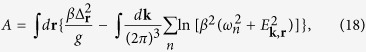


with 
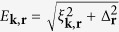
 being the BCS-Bogliubov excitation energy and *β* = 1/*k*_*B*_*T*. The gap Δ_**r**_ is determined by *δA*/*δ*Δ_**r**_ = 0:


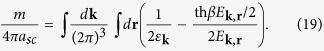


In the mean-field approximation, the total particle density distribution in the phase space is


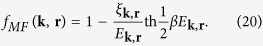


Then 
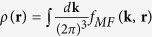
 and 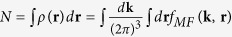 can be used to determine the chemical potential *μ*. Since the free energy is *F* = *A*/*β* and the entropy is *S* = −*k*_*B*_∂(*TA*)/∂*T*, the energy has the form


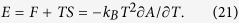


Then in order to calculate the viscous relaxation time *τ*_*ns*_ in the normal fermion scatting with superfluid pairs, we want to get the fermion-pair coupling constant *g*_*ns*_ in the order phase. As described above for getting the expression for *g*_*ns*_, we combine the one-channel microscopic theory with the two-liquid approach. We suppose that this system can also be described phenomenally as a mixture of normal fluid formed by *N*_*n*_ fermions and superfluid formed by *N*_*s*_ pairs composed of two fermions in the whole regimes with the total atomic number conservation: *N* = *N*_*n*_ + 2*N*_*s*_. This is just the two-fluid model. For a trapped system, we have


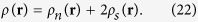


The effective energy density is 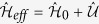, with 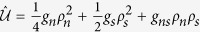 being the interaction density. Since we lack the knowledge of the expressions for *g*_*s*_ and *g*_*ns*_ in the one-channel microscopic model even at the mean-field level, we derive them in the phenomenological way. The mean superfluid pair-superfluid pair(normal fermion-normal fermion) interaction energy per particle is


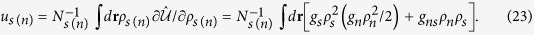


We consider the distribution function of normal fermion fluid in the phase space as a simple form


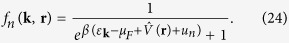


We take the same approximation as in the mean-field theory of the one-channel microscopic model, and assume the *N*_*s*_ pairs have no fluctuations and are all frozen at the ground state with **q** = 0. So only fermions in the normal fluid contribute to *K*. The kinetic energy is only from *N*_*n*_ fermions and the potential energy is from all *N* fermions:


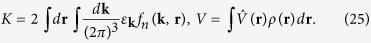


The total energy is


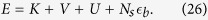


Here the interacting energy is 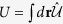 and the binding energy is 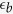. From the chemical equilibrium condition, we have *μ* = *μ*_*F*_ = *μ*_*B*_/2. Since 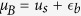, we have 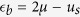. Because Δ_**r**_ → 0 when *r* → ∞, there is always *u*_*n*_ = 0. In this case, *g*_*n*_/*g*_*ns*_ is determined by


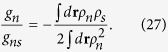


*g*_*s*_ is determined by equaling Eq. (21) to Eq. (26). So far all parameters are well-defined and we can use them to calculate *τ*_*nn*_,* τ*_*ns*_, (*K*, *V*, *U*) and further *ξ*_*i*_ and *ζ*_*i*_ for *i* = (⊥, *z*) in the order phase at *T* < *T*_*c*_. Since two viscous relaxation time *τ*_*nn*_ and *τ*_*ns*_ are within the Boltzmann-Vlasov kinetic theory, our approach is combined with the two-fluid physical picture and the one-channel microscopic theory. This theory is within both the *T*-matrix fluctuation theory in the normal phase and within the mean-field theory in the order phase.

Remarkably, this mean-field theory in the one-channel microscopic model is the simplest theory to study the BCS-BEC crossover system at *T* < *T*_*c*_. It catches the main characteristics of the system and includes the pairing and depairing effects. Strictly speaking, the mean-field theory only makes sense at the deep BCS side and BEC limit because it omits the pair fluctuations which play an important role near the unitary limit. However, we only use the Boltzmann-Vlasov kinetic theory to study the collective mode and damping rate in this work. The mean-field theory proves a qualitative correct picture at the BCS side where the phase transition locates and includes the pairing and depairing effects which is our main interest. So our theory makes sense for it combines the phenomenological Boltzmann-Vlasov kinetic theory with the mean-field theory at *T* < *T*_*c*_ to study the collective mode and damping rate including the pairing and depairing effects.

### Two viscous relaxation time of a trapped strongly interacting Fermi gas

We now calculate the viscous relaxation time. Take the trial function^68^ 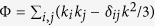, the denominator on the right side of Eq. (5) turns to


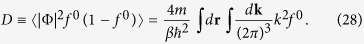


Here we have used the fact in the symmetry of the integral functions that 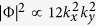. After the solid angle integral in the denominator, we have 
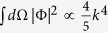
.

Under the Galilean invariance of the collision process, it is convenient to use the center-of-mass wave vector **k**_0_ and the relative wave vector coordinates **k**_*r*_ and 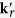 instead of the wave vectors of the incoming and outgoing particles **k**_1_, **k**_2_ and **k**_3_, **k**_4_:


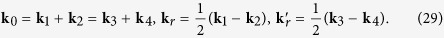


For atomic gases close to a Feshbach resonance under consideration in this work, a multichannel effective theory^20^,^73^ for atom-atom scattering must be taken into account. Under the broad band approximation close to a resonance, the particle-particle scattering cross section is


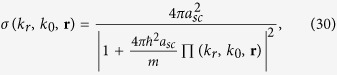


where Π(*k*_*r*_, *k*_0_, **r**) is the pair propagator and it is expressed on shell version as


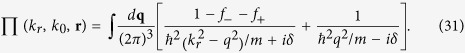


Here 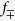 is the equilibrium Fermi distribution function *f  *^0^ with momentum 
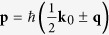
, and *δ* is an infinitesimal positive constant. In a vacuum, 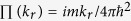, and 
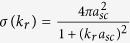
, as well known, is the unitarized vacuum scattering cross section.

At *T* ≥ *T*_*c*_, only *τ*_*nn*_ contributes to *τ*. This term is still important at *T* < *T*_*c*_. From^67^, the first numerator on the right side of Eq. (5) turns to


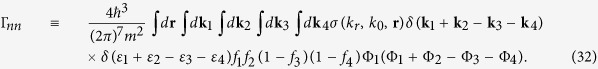


Using the symmetry of the collision integral under interchange of incoming and outgoing particle momenta, we may write 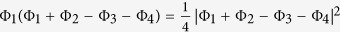. In the means of the integrals of the azimuthal angles of the vectors **k**_*r*_ and 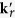, |Φ_1_ + Φ_2_ − Φ_3_ − Φ_4_|^2^ becomes proper to 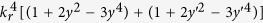 and *y*^4^ + *y*′^4^ becomes 2*y*^2^*y*′^2^. Here *y* = cos *θ*_*r*_ and 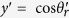 with *θ*_*r*_ and 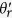 being the polar angles of the vectors **k**_*r*_ and 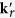. So that |Φ_1_ + Φ_2_ − Φ_3_ − Φ_4_|^2^ becomes proper to 1 + *y*^2^ + *y*′^2^ − 3*y*^2^*y*′^2^. The general dimensionless viscous fermion-fermion relaxation rate 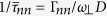 can be expressed as


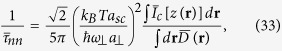


where 
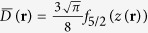
, 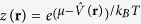 is the fugacity in the scattering, and 
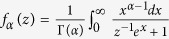
 is the Fermi function for *α* > 0. 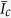 is the same as the one in^65^,^66^:


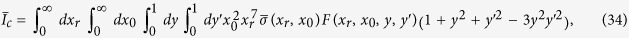


where 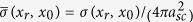 and 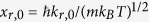 are all dimensionless. *F* is expressed as


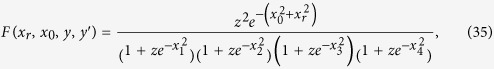


where the dimensionless variables 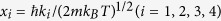 are the function of *x*_*r*_, *x*_0_, *y* and *y*′:


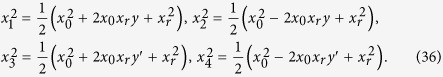


There are at least two channels contribute to *τ*_*ns*_. One is elastic with unbreakable pairs, the other is un-elastic with breakable pairs after scattering. The first channel only changes the momentum of fermions and fermion pairs, and it is important to *τ*_*ns*_ at *T* ≪ *T*_*c*_. It will complicates the calculations duo to the pair fluctuations. The second channel changes the momentum and energy of fermions as well as the fermion’s particle numbers of the normal fluid or superfluid, and it contributes to *τ*_*ns*_ near *T*_*c*_. In this work we only consider the second channel and write the second numerator on the right side of Eq. (5) to


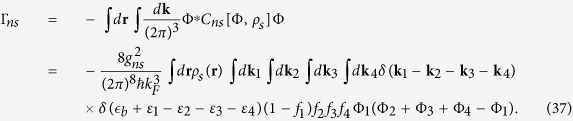


Here the incoming particles are a fermion pair with zero momentum and energy 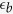 and a fermion with wave vector **k**_1_ and energy *ε*_1_, while the outgoing particles are three fermions with wave vectors **k**_*i*_ and energies *ε*_*i*_ for *i* = 2, 3, 4. Since the kinetic energies do not conserve due to 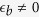 in the scatting process, we can’t easily choose a center-of-mass frame to simplify the calculations of delta function. The conversions of the momentum and energies in the scattering processes are expressed as


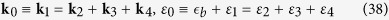


In the multidimensional momentum integral of Eq. (37), according to the symmetry of the integral function, the factor Φ_1_(Φ_2_ + Φ_3_ + Φ_4_ − Φ_1_) is proper to 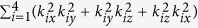. We first perform the momentum integral 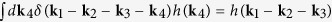 for a given function *h* in Eq. (37). We then choose the first wave vector elements 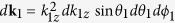 in spherical polar coordinates (*k*_1*z*_, *θ*_1_, *ϕ*_1_) with the radial wave vector fixed along the *z*–axial *k*_1*z*_ in Cartesian coordinates; the second wave vector elements *d***k**_2_ = *k*_2*x*_*dk*_2*x*_*dϕ*_2_*dk*_2*z*_ in cylindrical coordinates with plane polar coordinates (*k*_2*x*_, *ϕ*_2_) and *z*–axial coordinate *k*_1*z*_; and the combination 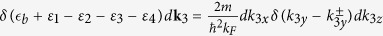 for the third wave vector elements in the Cartesian coordinates (*k*_3*x*_, *k*_3*y*_, *k*_3*z*_), where 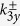 is determined by Eq. (38) of the conservation of the energies in the scattering processes. The denominator is the same as that in *τ*_*nn*_. The general dimensionless viscous fermion-pair relaxation rate is 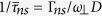 and the numerator is written as


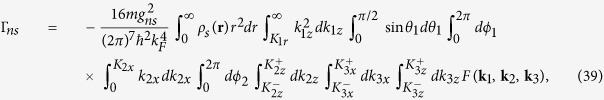


where 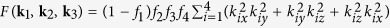 with **k**_4_ = **k**_1_ − **k**_2_ − **k**_3_ in Eq. (38). Note that 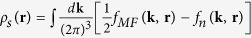 combines the mean-field theory with the two-fluid mixture approach.

The integral limits of Eq. (39) are given below. Firstly, for large enough *k*_1*z*_ to both break the dimer and scatter them into three outgoing particles with wave vectors (**k**_2_, **k**_3_, **k**_4_), the minimum of the total energy *ε*_0_ requires both *k*_2*r*_ = *k*_3*r*_ = *k*_4*r*_ = *k*_1*r*_/3 and 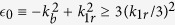. Therefore the minimum *k*_1*r*_ is 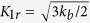, else the scattering process is forbidden. In the same method, 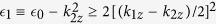 and the up and down integral limits of *k*_2*z*_ are 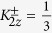 
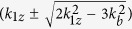
. From 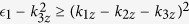, we also have 
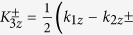
 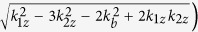. Secondly, since 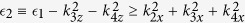 with the momentum conservation in the *x*–direction (*k*_4*x*_ = −*k*_2*x*_ − *k*_3*x*_) and 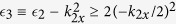, we can obtain the maximum 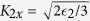, else the scattering process is also forbidden. From 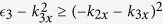, we have the up and down integral limits of *k*_3*x*_ as 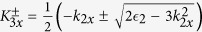. And finally, from the energy conservation 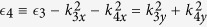 with the momentum conservation in the *y*–direction (*k*_3*y*_ = −*k*_4*y*_), we get the fixed 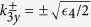. Consequently, the integral function *F*(**k**_1_, **k**_2_, **k**_3_) and the integral limits are all given above and we can carry out the calculation of Γ_*ns*_ in Eq. (39).

### Comparison with experiments

**Figure 1:**
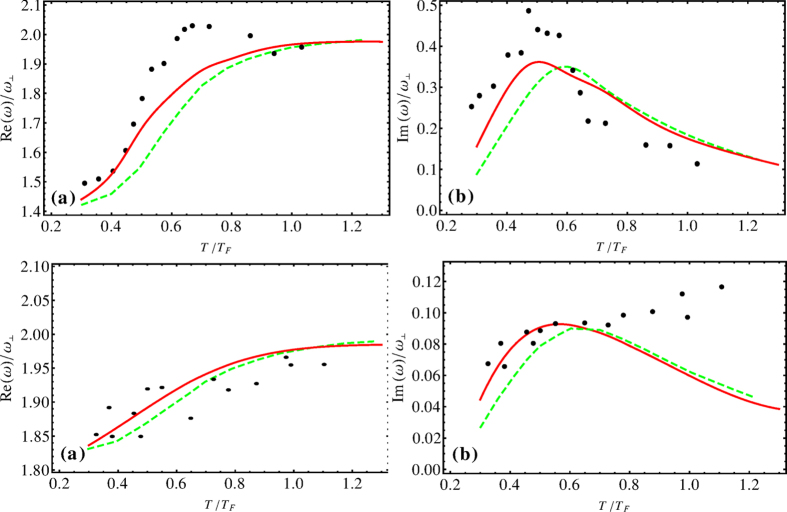
(**a**) Frequency Re*ω* and (**b**) damping Im*ω* in the units of *ω*_⊥_ versus the reduced temperature in the unit of Fermi temperature *T*_*F*_ for the quadrupole mode (upper plots) and compressions mode (lower plots) at the unitarity limit. The points are experimental values from^10^, while the red solid lines represent our computing results with many-body effects. The green dashed lines are^10^’s results with both Pauli blocking and pairing effects. Ours red solid lines conform better to the experimental results with the parameters *η* = −10^−3^, *N* = 6 × 10^5^, *λ* = 32/1800 (upper plots) and *λ* = 26/1100 (lower plots).

We have first used the *T*-matrix fluctuation theory to calculate the critical temperature *T*_*c*_ as a function of the interaction strength. With the experimental parameters, we then have calculated the gap Δ, coupling constants *g*_*s*_ and *g*_*ns*_ and density of bosons *ρ*_*s*_(**r**) below *T*_*c*_, and later the chemical potential *μ*, density of fermions *ρ*(**r**), ratios *ζ*_⊥_, *ξ*_⊥_, *ζ*_*z*_, and *ξ*_*z*_, index *γ*, and two viscous relaxation time *τ*_*nn*_ and *τ*_*ns*_, and so on, around *T*_*c*_ between the BCS and unitary limits. Finally, we have solved Eq. (11) with different physical parameters (*T*, *η*, *λ*, *N*). The following is our numerical results and discussions on the properties of the system. Numerical calculations show in Fig. 1 for the temperature dependent (a) frequency Re*ω* and (b) damping Im*ω* for the quadrupole mode (upper plots) and compressions mode (lower plots) at the unitarity limit, respectively. The curves are given for *T* ≥ *T*_*c*_ = 0.3*T*_*F*_. The red solid lines are the numerical results with the full scattering matrix in Eq. (31). As a comparison, we also show the experimental points and theoretical plots from^10^. Our results are essentially consistent with the experimental values.

**Figure 2:**
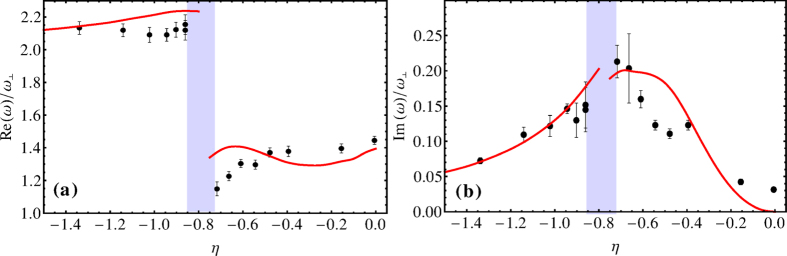
(**a**) Frequency Re*ω* and (**b**) damping Im*ω* in the units of *ω*_⊥_ versus the inverse reduced interaction strength *η* for the quadrupole mode near the phase transition. The points are experimental values from^1^,^14^, the red solid lines represent our computing results with many-body effects. Ours red solid lines conform to the experimental results with the parameters *T* = 0.1*T*_*F*_, *N* = 4 × 10^5^, *λ* = 22/370.

Figure 2 shows (a) frequency Re*ω* and (b) damping Im*ω* in the units of *ω*_⊥_ versus *η* at *T* = 0.1*T*_*F*_ for the quadrupole mode. The points are experimental values from^1^,^14^, while the red full lines represent our computing results. From Fig. 2, we can see that *η* around 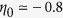, the frequency exhibits a pronounced jump from the hydrodynamic (*η* > *η*_0_) to the collisionless (*η* < *η*_0_) frequency due to the maximum of 1/*τ*_*ns*_ at *T* ≤ *T*_*c*_ 74. This transition is accompanied by a pronounced maximum of the damping rate. This is a striking transition from hydrodynamic to collisionless behavior and it comes from the minimum of *τ*_*ns*_ at *T*_*c*_. The present work provides strong evidence that quasistatic hydrodynamic theory does not apply to collective modes of a strongly interacting fermionic superfluid, when the oscillation frequencies approach the pairing gap. The sharp transition occurs at the maximum of viscous relaxation rate of normal fermion-superfluid pair collision at phase transition point. Above this point the pairing gap is breaking. Meanwhile, the strong damping is due to the fast varying of the frequency of collective modes along the BCS-unitarity crossover.

From the BCS limit to the left of the shaded region in Fig. 2, the system is at the normal phase and behaves as a collisionless Fermi gas at *T* > *T*_*c*_; while from the unitarity limit to the right of the shaded region, the system is at the order phase and behaves as superfluid fermion pairs at *T* < *T*_*c*_. Inside the shaded region in Fig. 2 both above approaches for *η*_*i*_ = 0 and *η*_*i*_ ≠ 0 are not applicable with *η*_*i*_ = 1 − *ζ*_*i*_ − *ξ*_*i*_ for *i* = (⊥, *z*). In this region the system is neither normal phase nor superfluid phase, which is a critical region and the system will have complex behaviors.

**Figure 3:**
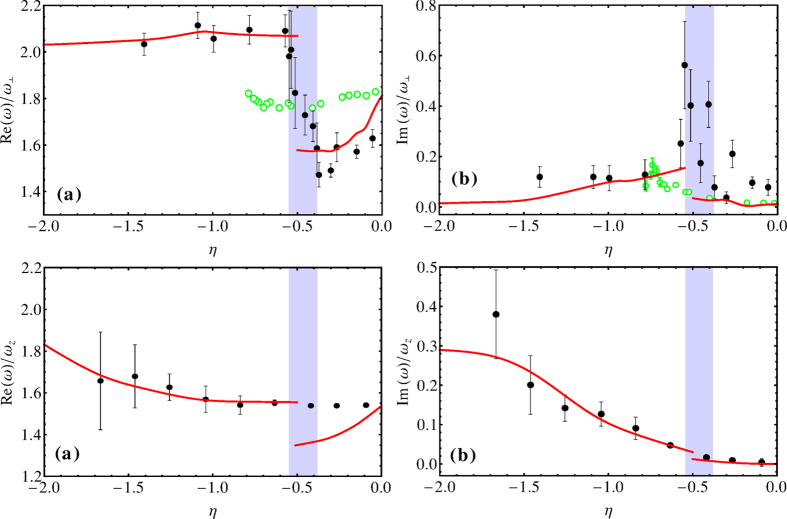
(**a**) Frequency Re*ω* and (**b**) damping Im*ω* in the units of *ω*_⊥_ (upper plots) and *ω*_*z*_ (lower plots) versus the inverse reduced interaction strength *η* for the radial compression mode (upper plots) and axial compression mode (lower plots) near the phase transition. The points are experimental values from^8^, the red solid lines represent our computing results with many-body effects, with the parameters *T* = 0.085*T*_*F*_, *N* = 4 × 10^5^, the radial vibration frequency *ω*_⊥_ = 2*π* × 750 Hz, and the axial vibration frequency 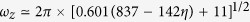 Hz. Our red solid lines conform better to the experimental results^8^. The green circles in the upper plots are experimental values from^6^, different both from the experimental values of 8 or our red solid lines, except near the unitarity limit.

Figure 3 shows (a) frequency Re*ω* and (b) damping Im*ω* versus *η* for the radial compressions mode (upper plots) and axial compression mode (lower plots). In the regime of a strongly interacting Fermi gas, an abrupt change in the collective excitation frequency occurs, we show that it is a signature of a transition from a superfluid to a collisionless phase. The measurements on the radial compression mode show three surprises^8^. The abrupt change of the excitation frequency and the large damping rate are not expected in a normal degenerate Fermi gas, where the collective excitation frequency is expected to vary smoothly from the hydrodynamic regime to the collisionless one. Furthermore, for the damping rate of the radial compressions mode in the transition regime, a maximum value is 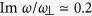. The measured damping rate of 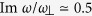 is clearly inconsistent with our prediction for the normal hydrodynamic regime. Of course, for the experimental parameters of *T* = 0.1*T*_*F*_, *N* = 2 × 10^5^, and *λ* = 70/1500 from^6^, the transition occurs at *η*_0_ = 0.79 with a smooth value of frequency Re*ω* and a maximum damping rate of Im*ω* = 0.2. Anyway, both the sudden change of the collective frequency and a strong damping may due to a transition from the superfluid phase to the normal phase and we need to study the superfluid phase in more details.

In summary, we have determined the transition and damping of collective modes in a trapped Fermi gas near the unitarity limit, based on the Boltzmann-Vlasov kinetic equation, combined with the many-body *T*-matrix fluctuation approximation in the normal phase and the many-body mean-field approximation in the order phase, and joined the two-fluid approach to connect the microscopic and phenomenal theories. We have calculated the dependence of temperature and scattering length on the frequency and damping of the collective modes, by using both theoretical and available experimental knowledge of the equation of state and two theoretical viscous relaxation time with the collision probability of fermion-fermion scatting with and without fermion pairs, including a many-body scattering effect. Our results agree well with the experimental observations, not only qualitatively but also quantitatively, particularly for the sharp transition and strong damping of the collective modes in the BCS side near the phase transition when breaking the pairing gap. This theory provides a link between the microscopic and kinetic descriptions of many-body Feshbach scattering. Notes that we have not considered the superfluid dynamics below *T*_*c*_, and omit the pair fluctuations which have the important effects near the Feshbach resonance. We may propose a new theory valid in the whole regime by adding these aspects further.

## Additional Information

**How to cite this article**: Dong, H. *et al.* Transition and Damping of Collective Modes in a Trapped Fermi Gas between BCS and Unitary Limits near the Phase Transition. *Sci. Rep.* **5**, 15848; doi: 10.1038/srep15848 (2015).

## References

[b1] Giorgini S., Pitaevskii L. P. & Stringari S. Theory of ultracold atomic Fermi gases. Rev. Mod. Phys. 80, 1215 (2008).

[b2] Ketterle W. & Zwierlein M. W. Making, probing and understanding ultracold Fermi gases. Ultracold Fermi Gases, Proceedings of the International School of Physics Enrico Fermi, Course CLXIV Edited by Inguscio M., Ketterle W. & Salomon C. (IOS Press, Amsterdam, 2008).

[b3] Bloch I., Dalibard J. & Zwerger W. Many-body physics with ultracold gases. Rev. Mod. Phys. 80, 885 (2008).

[b4] Zwerger W. (Edit) The BCS-BEC Crossover and the Unitary Fermi Gas (Springer Verlag, 2012).

[b5] Kinast J. *et al.* Evidence for superfluidity in a resonantly interacting Fermi gas. Phys. Rev. Lett. 92, 150402 (2004). 15169270 10.1103/PhysRevLett.92.150402

[b6] Kinast J., Turlapov A. & Thomas J. E. Breakdown of hydrodynamics in the radial breathing mode of a strongly interacting Fermi gas. Phys. Rev. A 70, 051401(R) (2004).

[b7] Kinast J., Turlapov A. & Thomas J. E. Damping of a Unitary Fermi Gas. Phys. Rev. Lett. 94, 170404 (2005). 15904273 10.1103/PhysRevLett.94.170404

[b8] Bartenstein M. *et al.* Collective excitations of a degenerate gas at the BEC-BCS crossover. Phys. Rev. Lett. 92, 203201 (2004). 15169351 10.1103/PhysRevLett.92.203201

[b9] Altmeyer A. *et al.* Precision measurements of collective oscillations in the BEC-BCS crossover. Phys. Rev. Lett. 98, 040401 (2007). 17358745 10.1103/PhysRevLett.98.040401

[b10] Riedl S. *et al.* Collective oscillations of a Fermi gas in the unitarity limit: Temperature effects and the role of pair correlations. Phys. Rev. A 78, 053609 (2008).

[b11] Tey M. K. *et al.* Collective modes in a unitary Fermi gas across the superfluid phase transition. Phys. Rev. Lett. 110, 055303 (2013). 23414029 10.1103/PhysRevLett.110.055303

[b12] Hou Y.-H., Pitaevskii L. P. & Stringari S. First and second sound in a highly elongated Fermi gas at unitarity. Phys. Rev. A 88, 043630 (2013).

[b13] Wright M. J. *et al.* Finite-temperature collective dynamics of a Fermi gas in the BEC-BCS crossover. Phys. Rev. Lett. 99, 150403 (2007). 17995145 10.1103/PhysRevLett.99.150403

[b14] Altmeyer A. *et al.* Dynamics of a strongly interacting Fermi gas: The radial quadrupole mode. Phys. Rev. A 76, 033610 (2007).

[b15] Luo L. *et al.* Measurement of the entropy and critical temperature of a strongly interacting Fermi gas. Phys. Rev. Lett. 98, 080402 (2007). 17359072 10.1103/PhysRevLett.98.080402

[b16] Ku M. J. H., Sommer A. T., Cheuk L. W. & Zwierlein M. W. Revealing the superfluid Lambda transition in the universal thermodynamics of a unitary Fermi gas. Science 335, 563 (2012). 22245739 10.1126/science.1214987

[b17] Guéry-Odelin D. Mean-field effects in a trapped gas. Phys. Rev. A 66, 033613 (2002).

[b18] Massignan P., Bruun G. M. & Smith H. Viscous relaxation and collective oscillations in a trapped Fermi gas near the unitarity limit. Phys. Rev. A 71, 033607 (2005).

[b19] Kavoulakis G. M., Pethick C. J. & Smith H. Collisional relaxation in diffuse clouds of trapped bosons. Phys. Rev. A 61, 053603 (2000).

[b20] Bruun G. M. & Smith H. Viscosity and thermal relaxation for a resonantly interacting Fermi gas. Phys. Rev. A 72, 043605 (2005).

[b21] Bruun G. M. & Smith H. Frequency and damping of the scissors mode of a Fermi gas. Phys. Rev. A 76, 045602 (2007).

[b22] Bruun G. M. & Mottelson B. R. Low energy collective modes of a superfluid trapped atomic Fermi gas. Phys. Rev. Lett. 87, 270403 (2001). 11800862 10.1103/PhysRevLett.87.270403

[b23] Szymanska M. H., Goral K., Kohler T. & Burnett K. Conventional character of the BCS-BEC crossover in ultracold gases of ^40^K. Phys. Rev. A 72, 013610 (2005).

[b24] Sade Melo C. A. R., Randeria M. & Engelbrecht J. R. Crossover from BCS to Bose superconductivity: Transition temperature and time-dependent Ginzbeurg-Landau theory. Phys. Rev. Lett. 71, 3202 (1993). 10054883 10.1103/PhysRevLett.71.3202

[b25] Engelbrecht J. R., Randeria M. & Sade Melo C. A. R. BCS to Bose crossover: Broken-symmetry state. Phys. Rev. B 55, 15153 (1997).

[b26] Taylor E., Griffin A., Fukushima N. & Ohashi Y. Pairing fluctuations and the superfluid density through the BCS-BEC crossover. Phys. Rev. A 74, 063626 (2006).

[b27] Altland A. & Sinons B. Condensed Matter Field Theory (Cambridge University Press, Cambridge, 2008).

[b28] Bruun G. M. Universality of a two-component Fermi gas with a resonant interaction. Phys. Rev. A 70, 053602 (2004).

[b29] Nozières P. & Schmitt-Rink S. Bose condensation in an attractive fermion gas: From weak to strong coupling superconductivity. J. Low Temp. Phys. 59, 195 (1985).

[b30] Haussmann R. Properties of a Fermi liquid at the superfluid transition in the crossover region between BCS superconductivity and Bose-Einstein condensation. Phys. Rev. B 49, 12975 (1994). 10.1103/physrevb.49.12975 10010209

[b31] Ohashi Y. & Griffin A. BCS-BEC crossover in a gas of Fermi atoms with a Feshbach resonance. Phys. Rev. Lett. 89, 130402 (2002). 12225012 10.1103/PhysRevLett.89.130402

[b32] Ohashi Y. & Griffin A. Superfluid transition temperature in a trapped gas of Fermi atoms with a Feshbach resonance. Phys. Rev. A 67, 033603 (2003).

[b33] Ohashi Y. & Griffin A. Superfluidity and collective modes in a uniform gas of Fermi atoms with a Feshbach resonance. Phys. Rev. A 67, 063612 (2003).

[b34] Perali A., Pieri P., Pisani L. & Strinati G. C. BCS-BEC crossover at finite temperature for superfluid trapped Fermi atoms. Phys. Rev. Lett. 92, 220404 (2004). 15245204 10.1103/PhysRevLett.92.220404

[b35] Hu H., Liu X.-J. & Drummond P. D. Equation of state of a superfluid Fermi gas in the BCS-BEC crossover. Europhys. Lett. 74, 574 (2006).

[b36] Hu H., Liu X.-J. & Drummond P. D. Temperature of a trapped unitary Fermi gas at finite entropy. Phys. Rev. A 73, 023617 (2006).

[b37] Combescot R., Leyronas X. & Kagan M. Yu. Self-consistent theory for molecular instabilities in a normal degenerate Fermi gas in the BEC-BCS crossover. Phys. Rev. A 73, 023618 (2006).

[b38] Nussinov Z. & Nussinov S. Triviality of the BCS-BEC crossover in extended dimensions: Implications for the ground state energy. Phys. Rev. A 74, 053622 (2006).

[b39] Haussmann R., Rantner W., Cerrito S. & Zwerger W. Thermodynamics of the BCS-BEC crossover. Phys. Rev. A 75, 023610 (2007).

[b40] Rohe D. & Metzner W. Pair-fluctuation-induced pseudogap in the normal phase of the two-dimensional attractive Hubbard model at weak coupling. Phys. Rev. B 63, 224509 (2001).

[b41] Perali A., Pieri P., Strinati G. C. & Castellani C. Pseudogap and spectral function from superconducting fluctuations to the bosonic limit. Phys. Rev. B 66, 024510 (2002).

[b42] Pieri P., Pisani L. & Strinati G. C. Comparison between a diagrammatic theory for the BCS-BEC crossover and quantum Monte Carlo results. Phys. Rev. B 72, 012506 (2005).

[b43] Chen Q., Stajic J., Tan S. & Levin K. BCS-BEC crossover: From high temperature superconductors to ultracold superfluids. Phys. Rep. 412, 1 (2005).

[b44] Hu H., Liu X.-J. & Drummond P. D. Comparative study of strong-coupling theories of a trapped Fermi gas at unitarity. Phys. Rev. A 77, 061605(R) (2008).

[b45] Leggett A. J. Bose-Einstein Condensation and Cooper Pairing in Condensed Matter Systems (Oxford University Press, Oxford, 2006).

[b46] Diener R. B., Sensarma R. & Randeria M. Quantum fluctuations in the superfluid state of the BCS-BEC crossover. Phys. Rev. A 77, 023626 (2008).

[b47] Bulgac A., Drut J. E. & Magierski P. Spin 1/2 fermions in the unitary regime: A superfluid of a new type. Phys. Rev. Lett. 96, 090404 (2006). 16606247 10.1103/PhysRevLett.96.090404

[b48] Burovski E., Prokofev N., Svistunov S. & Troyer M. Critical temperature and thermodynamics of attractive fermions at unitarity. Phys. Rev. Lett. 96, 160402 (2006). 16712207 10.1103/PhysRevLett.96.160402

[b49] Tsuchiya S., Watanabe R. & Ohashi Y. Single-particle properties and pseudogap effects in the BCS-BEC crossover regime of an ultracold Fermi gas above *T*_*c*_. Phys. Rev. A 80, 033613 (2009).

[b50] Watanabe R., Tsuchiya S. & Ohashi Y. Superfluid density of states and pseudogap phenomenon in the BCS-BEC crossover regime of a superfluid Fermi gas. Phys. Rev. A 82, 043630 (2010).

[b51] Tsuchiya S., Watanabe R. & Ohashi Y. Pseudogap temperature and effects of a harmonic trap in the BCS-BEC crossover regime of an ultracold Fermi gas. Phys. Rev. A 84, 043647 (2011).

[b52] Heiselberg H. Collective modes of trapped gases at the BEC-BCS crossover. Phys. Rev. Lett. 93, 040402 (2004). 15323737 10.1103/PhysRevLett.93.040402

[b53] Manini N. & Salasnich L. Bulk and collective properties of a dilute Fermi gas in the BCS-BEC crossover. Phys. Rev. A 71, 033625 (2005).

[b54] Astrakharchik G. E., Combescot R., Leyronas X. & Stringari S. Equation of state and collective frequencies of a trapped Fermi gas along the BEC-unitarity crossover. Phys. Rev. Lett. 95, 030404 (2005). 16090724 10.1103/PhysRevLett.95.030404

[b55] Zhang W. Y., Ma C. R. & Ma Y. L. Phenomenological equation of state and density profiles of strongly interacting trapped superfluid Fermi gases with arbitrary atom numbers. J. Low Temp. Phys. 159, 525 (2010).

[b56] Luo L. & Thomas J. E. Thermodynamic measurements in a strongly interacting Fermi gas. J. Low Temp. Phys. 154, 1 (2009).

[b57] Zhou L., Ma C. R. & Ma Y. L. Ground state and quadrupole excitations of anisotropically trapped Fermi superfluids with arbitrary atom numbers in the BCSCBEC crossover. J. Phys. B 40, 4591(2007).

[b58] Yin J., Ma Y. L. & Huang G. Analytical expressions of collective excitations for trapped superfluid Fermi gases in a BCS-BEC crossover. Phys. Rev. A 74, 013609 (2006).

[b59] Ma Y. L. & Huang G. Bogoliubov excitations of trapped superfluid Fermi gases in a BCS-BEC crossover beyond the Thomas-Fermi limit. Phys. Rev. A 75, 063629 (2007).

[b60] Hu H., Minguzzi A., Liu X.-J. & Tosi M. P. Collective modes and ballistic expansion of a Fermi gas in the BCS-BEC crossover. Phys. Rev. Lett. 93, 190403 (2004). 15600814 10.1103/PhysRevLett.93.190403

[b61] Salasnich L. & Toigo F. Extended Thomas-Fermi density functional for the unitary Fermi gas. Phys. Rev. A 78, 053626 (2008).

[b62] Kim Y. E. & Zubarev A. L. Collective excitations of strongly interacting Fermi gases of atoms in a harmonic trap. Phys. Rev. A 72, 011603(R) (2005).

[b63] Cao C. *et al.* Universal quantum viscosity in a unitary Fermi gas. Science 331, 58 (2011). 21148347 10.1126/science.1195219

[b64] Cao C., Elliott E., Wu H. & Thomas J. E. Searching for perfect fluids: Quantum viscosity in a universal Fermi gas. New J. Phys. 13, 075007 (2011). 10.1126/science.1195219 21148347

[b65] Nikuni T. & Griffin A. Hydrodynamic damping in trapped Bose gases. J. Low Tem. Phys. 111, 793 (1998).

[b66] Uehling E. A. & Uhlenbeck G. E. Transyort phenomena in Einstein-Bose and Fermi-Dirae gases. I. Phys. Rev. 43, 552 (1933).

[b67] Nikuni T., Zaremba E. & Griffin A. Two-fluid dynamics for a Bose-Einstein condensate out of local equilibrium with the noncondensate. Phys. Rev. Lett. 83, 10 (1999).

[b68] Nikuni T. & Griffin A. Landau-Khalatnikov two-fluid hydrodynamics of a trapped Bose gas. Phys. Rev. A 63, 033608 (2001).

[b69] Pedri P., Guéry-Odelin D. & Stringari S. Dynamics of a classical gas including dissipative and mean-field effects. Phys. Rev. A 68, 043608 (2003).

[b70] Guery-Odelin D., Zambelli F., Dalibard J. & Stringari S. Collective oscillations of a classical gas confined in harmonic traps. Phys. Rev. A 60, 4851 (1999).

[b71] Al Khawaja U., Pethick C. J. & Smith H. Kinetic theory of collective modes in atomic clouds above the Bose-Einstein transition temperature. J. Low Temp. Phys. 118, 127 (2000).

[b72] Thouless D. J. Perturbation theory in statistical mechanics and the theory of superconductivity. Ann. Phys. (NY) 10, 553 (1960).

[b73] Bruun G. M., Jackson A. D. & Kolomeitsev E. E. Multichannel scattering and Feshbach resonances: Effective theory, phenomenology, and many-body effects. Phys. Rev. A 71, 052713 (2005).

[b74] Miyake K & Yamada K. Dynamics of Bose-Einstein condensate and two-fluid hydrodynamics. Prog. Theor. Phys. 56, 1689 (1976).

